# Effects of BET Surface Area and Silica Hydrophobicity on Natural Rubber Latex Foam Using the Dunlop Process

**DOI:** 10.3390/polym16213076

**Published:** 2024-10-31

**Authors:** Danvanichkul Assadakorn, Gongxu Liu, Kuanfa Hao, Lichen Bai, Fumin Liu, Yuan Xu, Lei Guo, Haichao Liu

**Affiliations:** 1 College of Electromechanical Engineering, Qingdao University of Science & Technology, Qingdao 266061, China; assadakorndan@gmail.com (D.A.); l18765487207@163.com (G.L.); jayhao0730@163.com (K.H.); 15684195159@163.com (L.B.); liufumin@qust.edu.cn (F.L.); 2 National Engineering Research Center of Advanced Tire Equipment and Key Materials, Qingdao University of Science & Technology, Qingdao 266061, China; 18792469648@163.com

**Keywords:** latex foam, silica, Dunlop process, hydrophobicity, BET surface area

## Abstract

To reinforce natural rubber latex foam, fumed silica and precipitated silica are introduced into latex foam prepared using the Dunlop process as fillers. Four types of silica, including Aerosil 200 (hydrophilic fumed silica), Reolosil DM30, Aerosil R972 (hydrophobic fumed silica), and Sipernat 22S (precipitated silica), are investigated. The latex foam with added silica presents better mechanical and physical properties compared with the non-silica foam. The hydrophobic nature of the fumed silica has better dispersion in natural rubber compared to hydrophilic silica. The specific surface area of silica particles (BET) also significantly influences the properties of the latex foam, with larger specific surface areas resulting in better dispersity in the rubber matrix. It was observed that exceeding 2 phr led to difficulties in the foaming process (bulking). Furthermore, higher loading of silica also affected the rubber foam, resulting in an increased shrinkage percentage, hardness, compression set, and crosslink density. The crosslink density increased from 11.0 ± 0.2 mol/cm^3^ for non-silica rubber to 11.6 ± 0.6 mol/cm^3^ for Reolosil DM30. Reolosil DM30 also had the highest hardness, with a hardness value of 52.0 ± 2.1 IRHD, compared to 45.0 ± 1.3 IRHD for non-silica foam rubber and 48 ± 2.4 IRHD for hydrophilic fumed silica Aerosil 200. Hydrophobic fumed silica also had the highest ability to return to its original shape, with a recovery percentage of 88.0% ± 3.5% compared to the other fumed silica. Overall, hydrophobic fumed silica had better results than hydrophilic silica in both fumed and precipitated silica.

## 1. Introduction

Natural rubber, sourced from rubber trees, is a versatile material known for its elasticity. It finds wide applications in diverse industries, including bedding, thermal insulation, and automotive components [1]. Various types of foam rubber exist, each tailored to meet specific needs. The manufacturing process of foam rubber is relatively straightforward, with fillers playing a pivotal role in shaping the properties of the final product. Common fillers include calcium carbonate, silica, and carbon black [2,3]. These fillers serve as primary reinforcing agents, contributing to the structural integrity and functional characteristics of rubber compounds. Calcium carbonate, silica, and carbon black each exhibit unique properties that influence the mechanical and physical attributes of the rubber, as well as its surface chemistry and coloration [2,4]. The choice of filler depends on the industry and the specific type of rubber. In the tire industry, for example, carbon black is the primary filler of choice due to its exceptional reinforcing properties and compatibility with rubber compounds [5]. The addition of carbon black significantly enhances the mechanical strength, wear resistance, and overall performance of rubber used in tire manufacturing [6,7]. In the production of foam rubber, the utilization of fillers takes a different trajectory. Calcium carbonate stands out as a cost-effective and highly compatible filler. Its widespread use is attributed to its affordability and versatility, making it a preferred choice in various formulations. Similarly, silica plays a crucial role in shaping the characteristics of foam rubber. Beyond its reinforcing capabilities, silica introduces specific surface interactions, altering the material’s properties and surface chemistry [8].

Silica is an oxide of silicon with the chemical formula SiO_2_, commonly presented as a white powder. Its polar structure results in strong filler–filler interactions and adsorption [8,9]. As non-black fillers, silica performs well as a rubber reinforcing filler. Generally, carbon black reinforcement provides a higher modulus than silica reinforcement, but silica-reinforced rubber excels in several aspects, such as aging resistance, abrasion resistance, and adhesion properties [10,11]. Synthetic amorphous silica can be primarily categorized into two types: precipitated silica and fumed silica. The differentiation between these two variants lies in their distinct manufacturing processes and particle dimensions. Fumed silica, produced through the hydrolysis of silicon tetrachloride in a flame, is inherently hydrophilic due to its method of synthesis. The high surface area and fine particle size of fumed silica contribute to its excellent moisture absorption properties [12]. Hydrophilic fumed silica is particularly advantageous in applications requiring moisture absorption, such as in pharmaceuticals, cosmetics, and certain adhesive formulations [13,14]. Recognizing the demand for hydrophobic characteristics, surface modifications are often employed in the production of hydrophobic fumed silica. The surface treatment involves the introduction of hydrophobic functionalities, enhancing fumed silica’s resistance to moisture absorption, expanding the range of applications, improving dispersion, impacting rheological properties, and tailoring surface chemistry [15,16]. By contrast, precipitated silica is typically hydrophilic in its natural state. The production process of precipitated silica involves the acid precipitation of sodium silicate, resulting in a material with a hydrophilic surface [17]. Unlike fumed silica, which can be inherently hydrophilic or hydrophobic depending on its production process and subsequent surface treatments, the particle dimensions of precipitated silica are influenced by the specific conditions during precipitation. This type of synthetic silica has found diverse applications owing to its versatility and cost-effectiveness [17,18].

To the best of our knowledge, fumed and precipitated silica have not been widely used as fillers in the production of natural foam rubber using the Dunlop method. However, some researchers have explored the use of fumed silica and precipitated silica as fillers in the rubber and polymer industry. Bayat and Fasihi (2019) [19] studied the effect of coupling agents in natural rubber silica composite foam. They used different contents of precipitated silica and found that it was well-dispersed in natural rubber. The results showed that as the silica concentration increased, the density also increased, while the cell size decreased with higher silica content. Luo et al. [20] investigated the interaction between fumed silica and epoxidized natural rubber. Their research indicated that fumed silica had a strong interaction with epoxidized natural rubber and effectively contributed to crosslink density. Prasertsri et al. [21] studied the reinforcement of natural rubber using fumed silica and precipitated silica mixed by two-roll mills. The results showed that the samples using fumed silica as a filler had higher hardness, stiffness, and tensile strength compared to those using precipitated silica with the same content. However, the heat build-up in the samples containing fumed silica was significantly higher, indicating that the use of fumed silica as a filler must also consider the application requirements of the final product.

The aim of this present work is to study the reinforcement of natural rubber latex foam using fumed silica and precipitated silica. Latex foams were produced using different types of silica, including fumed silica (both hydrophilic and hydrophobic varieties) and precipitated silica. Zaborski et al. [12] highlighted the importance of considering the surface area of silica particles. Consequently, the effect of specific surface area of silica particles (BET) was also considered. The crosslink density, mechanical properties, as well as surface morphologies of latex foam were investigated to gain further insights into the reinforcing mechanism of silica in latex foam.

## 2. Experiment

### 2.1. Material

Table 1 displays the materials used in this work. Natural rubber latex with a high ammonia concentration (60% dry rubber content) was provided by Sri Trang Agro-Industry Public Company Limited (“STA”) (Surat Thani, Thailand). Three types of fumed silica and one type of precipitated silica were obtained from Evonik Degussa Co., Ltd. (Essen, Germany). Specifically, these included hydrophilic fumed silica (Aerosil 200, purity > 99.8%, BET surface area 200 ± 25 m^2^/g) and hydrophobic fumed silica (Aerosil R972, purity > 99.8%, BET surface area 110 ± 20 m^2^/g). Hydrophobic fumed silica (Reolosil DM30, purity > 99.8%, BET surface area 230 ± 20 m^2^/g) was purchased from Tokuyama Chemicals (Zhejiang) Co., Ltd. (Jiaxing, China). The hydrophilic precipitated silica (Sipernat 22s, purity > 97%, BET surface area 190 m^2^/g) was also purchased from Evonik Degussa Co., Ltd. (Essen, Germany). All silica samples were sourced from the same suppliers to ensure consistency; however, Tokuyama Chemicals did not have the specific silica specifications required for this study. Consequently, three types of silica were obtained from Tokuyama Chemicals and one type from Evonik Degussa Co., Ltd. (Essen, Germany), which met the specifications for pH, tamped density, and other critical parameters. Key characteristics of silica samples are displayed in Table 2. Sulfur was used as a vulcanizing agent and various additives were used, such as zinc oxide (ZnO_2_), potassium, zinc-N-diethyldithiocarbamate, zinc 2-mercaptobenzothiazole, Wingstay L, diphenyl guanidine, and sodium silicofluoride. These chemicals were prepared by Qingdao Amita Natural Latex., Co. Ltd. (Qingdao, China).

### 2.2. Rubber Compounds and Foam Preparation

**Figure 1:**
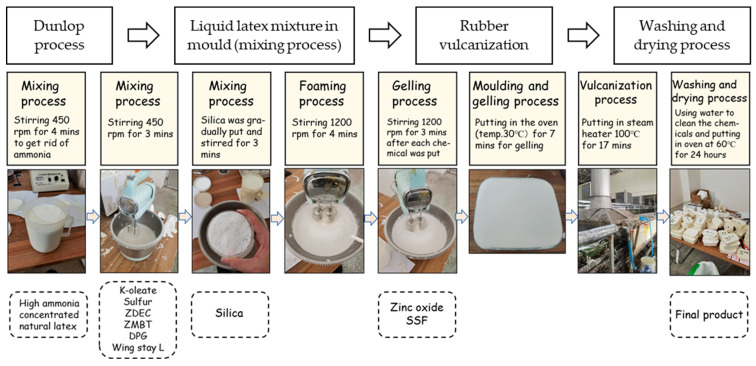
Schematic diagram of the Dunlop process.

Figure 1 shows the schematic diagram of the foaming process using the Dunlop method. The NR compound was prepared by using a stirring machine to mix the components as in Table 1 with a mechanical stirring speed of 450 rpm at room temperature of 15 °C. This initial low speed was chosen to ensure homogeneous mixing without risking premature vulcanization.

Mixing process: Natural rubber was added into the stirring machine, stirred for 4 min to remove the ammonia, which is essential for improving the viscosity and stability of the latex and facilitating better foam expansion. Afterward, the other ingredients consisting of K-oleate, sulfur, zinc Diethyldithiocarbamate, zinc 2-mercaptobenzothiazole, Wingstay L, and diphenyl guanidine were added and stirred for 3 min. These ingredients were included to enhance the crosslinking and stability of the rubber matrix during the vulcanization process.

Silica concentration: Silica was gradually added, while continuously stirring for 3 min. The concentration of each type of silica was controlled by adding four levels of content: 0.5 phr, 1 phr, 1.5 phr, and 2 phr. This range was selected to systematically assess the effects of varying silica concentrations on the foam’s mechanical properties. Concentrations exceeding 2.5 phr were avoided based on preliminary experiments conducted with this specific formulation, which indicated that higher silica content resulted in poor mixing and the onset of pre-vulcanization. This behavior compromised the uniformity and performance of the rubber compound. It is important to note that these observations are specific to the formulation used in this study, and other rubber formulations may exhibit different behaviors under similar conditions. Therefore, while the selected silica concentrations are appropriate for this particular system, further research may be necessary to explore the optimal silica levels for different formulations.

Foaming process: After mixing all these ingredients, the stirring speed was increased to 1200 rpm and the mixture was stirred for 4 min during the foaming process. This higher speed facilitates the uniform distribution of air bubbles, crucial for achieving the desired foam structure. The gelling agent, zinc oxide, was added and stirred for 3 min, and then Sodium silicofluoride dispersion was added and stirred for 2 min, respectively. The total mixing time for all samples was set to 19 min.

Temperature control: To mitigate the risk of premature vulcanization and gelation associated with heat generation, the room temperature was maintained at 15 °C throughout the mixing process. This temperature control helps to maintain the stability of the latex and prevent premature curing, which could otherwise adversely affect the foam structure.

Gelling process: After mixing all the materials according to the formulation in Table 1, foam rubber was put in the rectangular glass mold of 13 × 19 × 9 cm^3^, and the foam sample was placed in the oven at a temperature of 30 °C for 7 min as a gelling process. This gelling temperature was selected to promote the initial setting of the foam structure without triggering full vulcanization, which is essential for maintaining the desired expansion characteristics

Vulcanization process: Curing of samples was conducted at 100 °C for 17 min in a steam oven. This temperature and time were selected based on preliminary studies and experimental trials that demonstrated optimal crosslinking and mechanical performance under these conditions. These parameters were fine-tuned to ensure effective vulcanization, as the experiments indicated that this duration allowed for the best balance between curing and maintaining the desired material properties.

Washing and drying process: All samples after curing were washed with deionized water and placed in the oven at 60 °C for 24 h for drying.

### 2.3. Characterizations

#### 2.3.1. Foam Density

Foam densities were determined by cutting samples into 30 mm × 30 mm × 30 mm, according to ASTM D3574 [22]. The density was calculated using the mass and volume by the following equation:

(1) $$\rho_{f}=\frac{m_{f}}{v_{f}}$$

where, $m_{f}$ is the mass of the foam sample and $v_{f}$ is the volume.

#### 2.3.2. Crosslink Density

The samples were cut to a small size (4 × 2 × 25 mm^3^) for a crosslink density test based on the equilibrium swelling method. The crosslink density was calculated by using the Flory–Rehner equation [23,24]:

(2) $$-ln(1-V_{r})-V_{r}-\chi v_{r}^{2}=v_{s}\eta_{swell}\left(v_{r}^{1/3}-\frac{v_{r}}{2}\right)$$

where $V_{r}$ is the volume fraction of rubber in swollen gel, χ is the rubber–solvent interaction parameter, $v_{s}$ is the molar volume of toluene (106.8 cm^3^/${mol}^{-1}$), $\eta_{swell}$ is the swelling of the compounded rubber (mol/${cm}^{-3}$).

$V_{r}$ was tested according to ISO 1817 [25]. Samples were weighed and then swollen in toluene for 1 week. Then, the weights of the samples were measured, and the liquid on the surface of the samples was removed using filter paper. The samples were then dried at a temperature of 80 °C for 48 h. The weight after drying was measured and used to calculate the volume fraction of rubber in the swollen gel using the following equation [26,27]:

(3) $$V_{r}=\frac{\left[w_{0}\emptyset \frac{1-\alpha }{\rho_{r}}\right]}{\left[\left(w_{0}\emptyset \frac{1-\alpha }{\rho_{r}}\right)+\frac{\left(w_{1}-w_{2}\right)}{\rho_{s}}\right]}$$

(4) $$\alpha =\frac{w_{1}-w_{0}}{w_{1}}$$

(5) ∅=1−α

where $w_{0}$, $w_{1}$ are the weights (g) of samples before and after immersed in toluene, respectively, ∅ is the mass fraction of natural rubber (amount of natural rubber/total quantity), *α* is the mass fraction loss during swelling in toluene, and $\rho_{s}$ and $\rho_{r}$ are the densities (g/${cm}^{3}$) of toluene and the rubber composites, respectively.

(6) $$\chi =0.34+V_{s}{\left(\delta_{s}-\delta_{p}\right)}^{2}/RT$$

where $\delta_{s}$ and $\delta_{p}$ are the solubility parameters of toluene (8.26) and natural rubber, which are 8.26 and 8.91 ${cal}^{0.5}/{cm}^{1.5}$, respectively. R is the gas constant (cal/mol-K) and T is the absolute temperature (K) [28,29].

#### 2.3.3. Microstructural Analysis Using Scanning Electron Microscope (SEM)

The foam morphologies of the samples were determined using a scanning electron microscope (SEM). The samples were first surface-coated with gold using a sputter coater to ensure good electrical conductivity between the samples and the aluminum stub, thus preventing any charging effect during the observation. The SEM used was JEOL JSM-7500F (Tokyo, Japan). The acceleration voltage was set at 5 kV, and magnifications of ×50 and ×10,000 were used to observe the pore structure of the latex foam and the distribution of silica filler in the rubber matrix. The average diameter of cells was measured using ImageJ software (version 1.54g) from at least 300 different pores.

#### 2.3.4. Fourier Transform Infrared Spectroscopy Analysis (FTIR)

The chemical functional groups of the foam rubber samples were determined using attenuated total reflection Fourier transform infrared (ATR-FTIR) spectroscopy with a VERTEX 70 FT-IR spectrometer (Waltham, MA, USA). The foam rubber samples, sized 10 mm square with a 10 mm thickness, were placed on a Ge crystal probe and analyzed in the wave number range of 4000–600 cm^−1^.

#### 2.3.5. Compression Set

**Figure 2:**
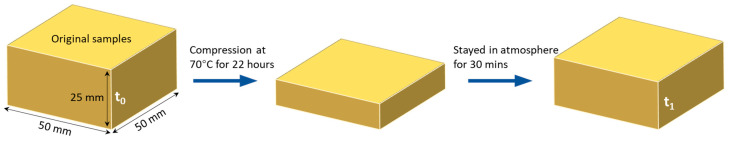
Schematic diagram for testing the compression set.

In the compression test according to the ASTM D3574 standard, the specimen size was 50 ± 1 mm (2-inch) square with a thickness of 25 ± 1 mm (1 inch). The samples were compressed by using a force transducer to reduce the thickness of the original sample to 50% and placed in an oven at a temperature of 70 °C ± 1 °C for 22 h, then the samples were taken out of the oven and the transducer and stayed in the atmosphere for 30 min. Then, the thickness of samples was measured. Figure 2 shows the schematic diagram for the test of the compression set. Three samples of each type were tested and the average was reported. The thickness measured was calculated by using the following two equations [30,31]:

(7) $$C_{d}=\left[\left(t_{0}-t_{1})/t_{0}\right.\right]\times 100$$

(8) $$recovery\;percentage=\frac{t_{1}}{t_{0}}\times 100$$

$C_{d}$ is the calculated percentage expressing the permanent deformation in relation to the total deformation, accounting for the transducer height (%). $t_{0}$ is the original height or thickness of the foam sample before compression, $t_{1}$ is the height or thickness of the foam sample after compression.

#### 2.3.6. Hardness Test

A hardness test was conducted using the International Rubber Hardness Degree (IRHD) tester, specifically the Wallace H14 Macro IRHD tester (Dorking, UK), in accordance with ASTM D1415 standards [32]. Specimens were precision-cut to dimensions of 10 mm in thickness, forming squares with sides measuring 20 mm at a controlled room temperature of 23 °C. For each specimen, hardness measurements were taken at five distinct points distributed across the surface. The median value of these measurements, rounded to the nearest IRHD, was calculated to determine the final hardness value. This approach ensures a representative and accurate characterization of the material’s hardness based on multiple points on each specimen.

#### 2.3.7. Percentage Shrinkage

The percentage shrinkage was determined in accordance with the standard of ASTM D1055 [33] by measuring the dimensions of the sample. All sides, including width, length, height, and the height of the center, were measured. The percentage shrinkage was calculated by comparing the changes in these dimensions between the sample after vulcanization and the dimensions of the mold. The equation below was used to calculate the percentage shrinkage:

(9) $$\mathrm{Percentage}\;\mathrm{shrinkage}=\left(\frac{X_{1}-X_{2}}{X_{1}}\right)\times 100$$

where $X_{1}$ = dimension of sides (cm), $X_{2}$= dimensions of sides of rubber after vulcanization.

## 3. Discussion and Results

The experiment was divided into two major parts for analysis. The first part investigated different types of silica, including fumed and precipitated silica, with a focus on both hydrophilic and hydrophobic varieties, as well as particle size. These factors were found to influence foam morphology. The second part studied the impact of silica content on the vulcanization of the latex foam produced using the Dunlop process.

### 3.1. Microstructural Analysis

**Figure 3:**
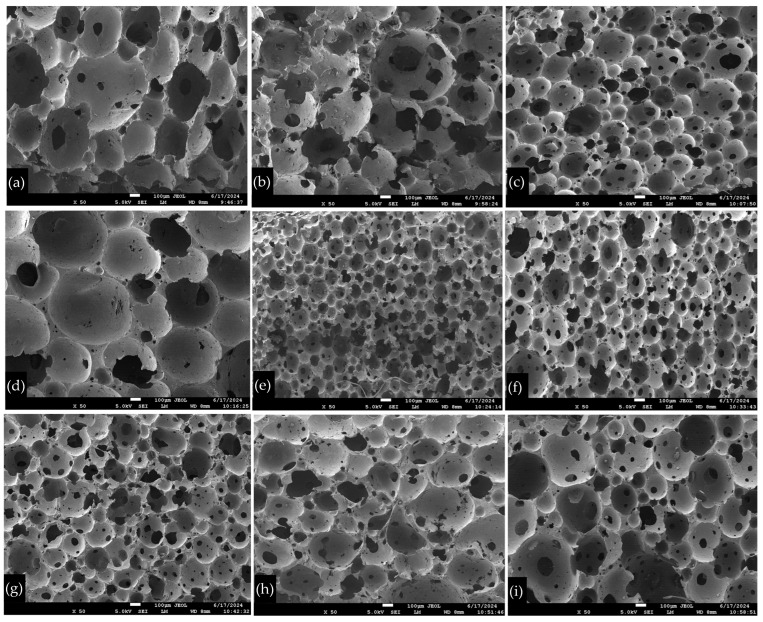
SEM image of natural latex foam (**a**) without silica, (**b**) with DM30 at 1 phr, (**c**) with DM30 at 2 phr, (**d**) with Aerosil R972 at 1 phr, (**e**) with Aerosil R972 at 2 phr, (**f**) with Aerosil 200 at 1 phr, (**g**) with Aerosil 200 at 2 phr, (**h**) with Sip22s at 1 phr, and (**i**) with Sip22s at 2 phr. SEM images of foam rubber at 50× magnification. A 100 µm scale bar is included for reference.

The surface morphologies of the latex foams were examined using SEM, as shown in Figure 3. The figure illustrates the structures of the foam samples produced by different types and concentrations of silica. According to Figure 3, it is evident that a higher concentration of silica results in a smaller cell size and a more uniform distribution of the cell structure. At a concentration of 1 phr, the SEM results demonstrate that the BET specific surface area and hydrophobicity/hydrophilicity of the silicas play a decisive role in determining the foam microstructure. Latex foams containing fumed hydrophobic silicas, such as Reolosil DM30 and Aerosil R972, exhibited a mixture of larger and smaller pore sizes; while fumed hydrophilic silica (Aerosil 200) and precipitated silica (Sipernat 22S) produced predominantly medium and smaller pore sizes (Figure 3). Increasing the silica concentration to 2 phr made the distinctions between the silica types less pronounced, as all the silicas were able to effectively promote a finer and more uniform cellular structure in the latex foam [34,35]. After mixing with natural rubber, it increased the viscosity of the rubber mixture, which reduced the bubble size during the foaming process [36]. Furthermore, a higher concentration of silica increased the nucleation sites for bubble formation, leading to the formation of a larger number of smaller bubbles, rather than fewer larger ones [37,38].

**Figure 4:**
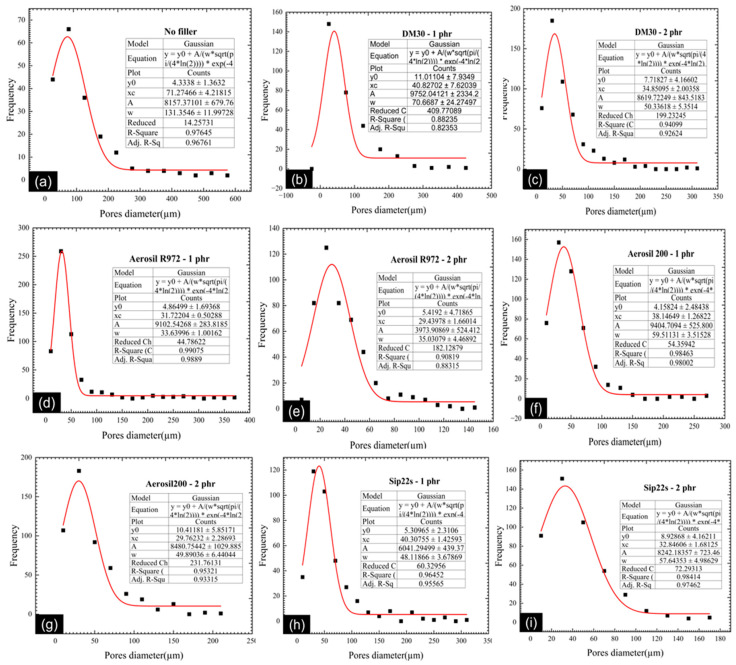
Average pores diameter of silicas (**a**) without silica, (**b**) with DM30 at 1 phr, (**c**) with DM30 at 2 phr, (**d**) with Aerosil R972 at 1 phr, (**e**) with Aerosil R972 at 2 phr, (**f**) with Aerosil 200 at 1 phr, (**g**) with Aerosil 200 at 2 phr, (**h**) with Sip22s at 1 phr, and (**i**) with Sip22s at 2 phr.

The ImageJ program was used to analyze the cell diameters, as shown in Figure 4. Fumed silica, Aerosil R972, has the smallest average pore diameters at both 1 phr and 2 phr concentrations. By contrast, fumed silica, Reolosil DM30, has the largest average pore diameter in both concentrations. These results suggest that particles with a larger BET specific surface area tend to produce larger average pore diameters. However, the hydrophilic fumed silica, Aerosil 200, which has nearly the same BET specific surface area as Reolosil DM30, resulted in smaller average pore diameters. Specifically, the average pore diameter of Aerosil 200 was 38.1 ± 1.3 μm at 1 phr and 29.8 ± 2.3 μm at 2 phr. With regard to Reolosil DM30, the average pore diameters were 40.8 ± 7.9 μm at 1 phr and 34.9 ± 2.0 μm at 2 phr. The precipitated silica, Sipernat 22S, which has a similar BET specific surface area, exhibited slightly different average pore diameters (40.3 ± 1.4 μm at 1 phr and 32.8 ± 1.7 μm at 2 phr) compared to hydrophobic fumed silica, Reolosil DM30.

There were slight differences between hydrophobic and hydrophilic types in the average pore diameters of silica. However, the pore structures of the foam rubber were distinct, as can be seen from Figure 3. While increasing the silica concentration resulted in smaller and more uniform cell sizes overall, there was notable variability between different silica types, particularly at 1 phr concentrations. Fumed hydrophobic silicas, such as Reolosil DM30, exhibited a broader range of pore sizes, reflected in larger standard deviations. By contrast, the hydrophilic fumed silica, Aerosil 200, and the precipitated silica, Sipernat 22S, produced more consistently sized, smaller pores.

**Figure 5:**
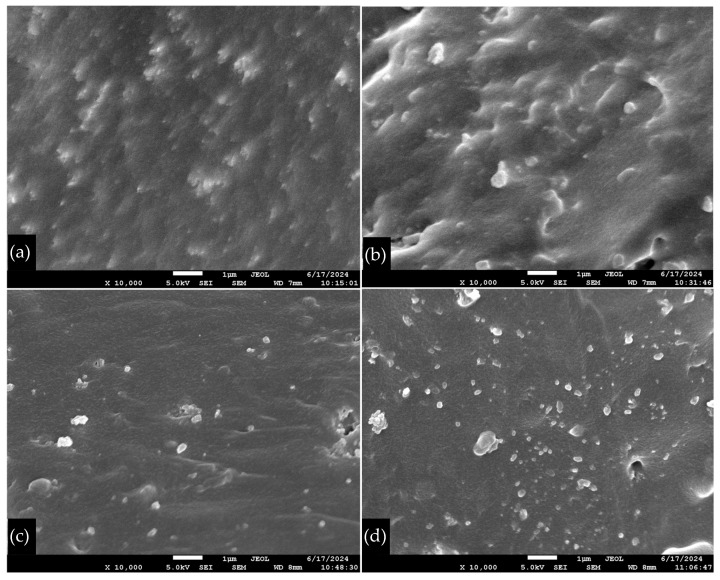
Morphology of the distribution of silicas in natural rubber. (**a**) DM30 at 2 phr, (**b**) Aerosil R972 at 2 phr, (**c**) Aerosil 200 at 2 phr, and (**d**) Sip22s at 2 phr. SEM images of foam rubber at 10,000× magnification. A 1 µm scale bar is included for reference.

From the SEM figures at 10,000× magnification, as shown in Figure 5, the hydrophobic fumed silica was observed to disperse more evenly within the natural rubber matrix compared to the hydrophilic fumed silica. The hydrophilic silica showed more pronounced aggregation, suggesting that hydrophobic silica had better compatibility with the natural rubber matrix [38]. Additionally, it can be seen that the BET surface area of the silica particles is a critical factor in their distribution within the rubber matrix. Silica particles with a larger BET surface area tended to have better dispersions [39].

Note: SEM images at 50× magnification were captured with a 100 µm scale bar in Figure 3 for accurate interpretation of the pore structures, while SEM images at 10,000× magnification were captured with a 1 µm scale bar in Figure 5 to provide detailed visualization of silica dispersion.

### 3.2. FTIR

**Figure 6:**
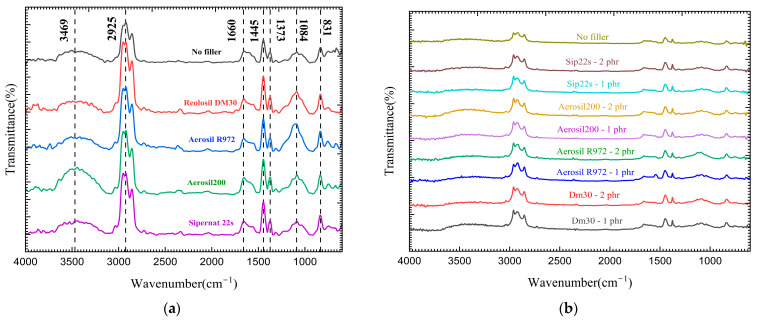
FTIR results of latex foam with different types of silica at 1 phr (**a**) and with different concentrations of silica (**b**).

ATR-FTIR spectroscopy was used to analyze the chemical interactions between natural rubber and different types and concentrations of silica (Figure 6). While the FTIR spectra did not reveal the presence of new functional groups across different silica types, variations in the intensity of existing peaks were observed, indicating possible physical interactions between silica and the rubber matrix. A band was observed at 1084 cm^−1^, corresponding to C–O–Si stretching vibrations. The presence of this band, even though subtle, suggests possible interactions between the silica particles and the natural rubber matrix [40]. These changes in peak intensity, rather than the appearance of new peaks, imply that the interactions are likely physical rather than chemical in nature at the tested silica concentrations.

Additionally, the peak intensity at 3469 cm^−1^, associated with the vibrations of hydroxyl groups (O-H), was notably higher in samples containing hydrophilic silicas, such as Aerosil 200 and precipitated, Sipernat 22S. This is consistent with their higher surface hydroxyl group density. Conversely, the hydrophobic silicas (Aerosil R972 and Reolosil DM30) exhibited a lower intensity in this region, which aligns with their lower hydroxyl group content. This distinction between hydrophilic and hydrophobic silicas reflects the expected behavior of these materials, where hydrophobic silicas have fewer surface hydroxyl groups due to their treatment to reduce moisture affinity.

The changes in peak intensities support the idea that silica interacts with the natural rubber matrix in a physical manner, affecting the overall dispersion of the silica within the rubber. The better dispersion of hydrophobic silicas, such as Aerosil R972 and Reolosil DM30, likely contributes to the improved mechanical properties observed in these samples. It is also important to note that FTIR’s sensitivity may not be sufficient to detect more subtle interactions at the low silica concentrations used in this study. Therefore, while no significant chemical bonding was detected between the silica and natural rubber, The observed variations in peak intensities at specific wavenumbers (1084 cm^−1^ and 3469 cm^−1^) indicate that physical interactions significantly influence the performance of the silica-filled rubber foam [41].

### 3.3. Foam Density

**Figure 7:**
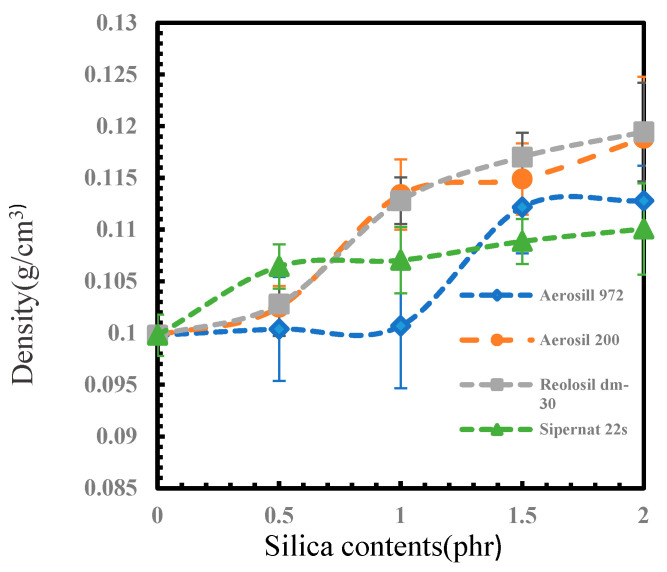
Density of different types of silica.

Figure 7 shows the densities of different types of silica with different contents. The density of the foam samples increased linearly with the increase in silica content for all types of silica. The highest density was observed for the samples containing 2 phr of Reolosil DM30 hydrophobic fumed silica (0.120 ± 0.005 g/cm^3^), followed by those with 2 phr of Aerosil 200 hydrophilic fumed silica (0.119 ± 0.006 g/cm^3^), then 2 phr of Aerosil R972 hydrophobic silica (0.113 ± 0.003 g/cm^3^), and finally 2 phr of Sipernat 22S precipitated silica (0.110 ± 0.004 g/cm^3^). The density of the non-silica foam rubber was 0.100 ± 0.002 g/cm^3^. The differences in density between the samples can be attributed to the ability of the silicas to create a finer and more uniform cellular structure, as observed in the SEM images. The higher the silica loading, the greater the reinforcement of the rubber matrix, leading to higher foam densities [42,43].

### 3.4. Crosslink Density

**Figure 8:**
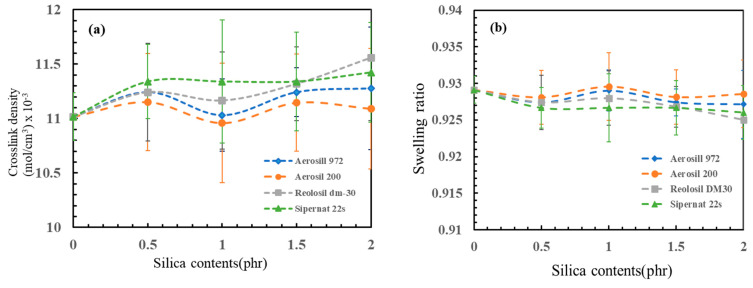
(**a**) Crosslink densities of different types of silica, (**b**) swelling ration of different types of silica.

The crosslink density of unfilled and silica-filled samples was estimated using the swelling method in toluene solvent, as shown in Figure 8. The swelling rate of samples with different silica loadings was nearly uniform, suggesting that the loading of silica had minimal influence on the swelling rate [44]. Despite slight differences in crosslink density, it can be observed that higher silica loadings corresponded to higher crosslink densities. The addition of silica promoted the dispersion of the vulcanization system in latex. During the mixing process of silica and latex, the fusion of rubber molecular chains with silica occurred, enhancing the crosslinking uniformity and efficiency [45].

At a silica concentration of 1 phr, the crosslink density was comparable to that of the non-silica sample. However, with increasing silica concentrations, crosslink density also increased. This is because the rubber cells decreased while the silica cells increased, leaving some silica cells unswollen. The crosslink density of non-silica foam rubber was (11.0 ± 0.2) × 10^−3^ mol/cm^3^. Among the types of silica, the fumed silica, Reolosil DM30, exhibited the highest crosslink density values (11.6 ± 0.6) × 10^−3^ mol/cm^3^ due to its high BET surface area and hydrophobic properties.

Hydrophobic silica particles, such as Reolosil DM30, repel water and have a higher affinity for non-polar solvents [46,47]. When incorporated into a rubber matrix, hydrophobic silica is less likely to interact with water molecules and may instead interact with non-polar segments of the rubber chains. This interaction promotes the formation of crosslinks between polymer chains during vulcanization, resulting in a higher crosslink density compared to systems without silica or with hydrophilic silicas [48].

Although the precipitated silica, Sipernat 22S, has a lower specific surface area, its higher bulk density compared to all three types of fumed silica can affect crosslink density [39]. A higher bulk density reduces cell space, resulting in higher silica cell concentrations. This suggests that the properties of silica, including surface chemistry and bulk density, play crucial roles in influencing crosslink density within rubber composites.

### 3.5. Compression Set

The compression set values, as shown in the above figures, varied with silica loading (0, 0.5, 1, 1.5 and 2 phr) across the four different types of silica. It was observed that the compression set values increased with higher silica loading. The hydrophobic fumed silica, Reolosil DM30, exhibited the highest values, followed by the hydrophobic fumed silica, Aerosil R972, the hydrophilic precipitated silica, Sipernat 22S, and finally the hydrophilic fumed silica, Aerosil 200.

**Figure 9:**
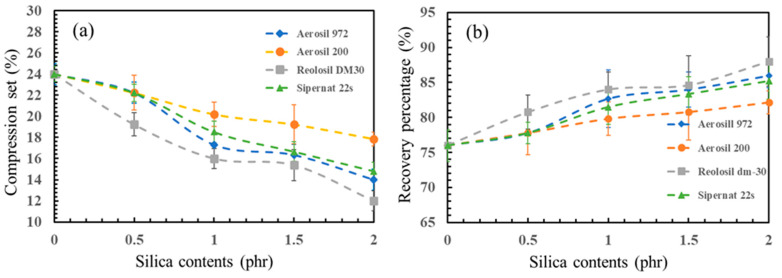
(**a**) Compression set of different types of silica; (**b**) recovery percentage of different types of silica.

Compression set values serve as an indicator of the elasticity of the latex foam, with lower values indicating higher elasticity and ability to return to its original shape [49]. The compression set value of the non-silica sample was the highest (24.0% ± 0.7%), due to the unevenly distributed pore characteristics within the rubber matrix [50,51]. By contrast, silica-reinforced latex foam exhibited a more even distribution of cells. The increased amount of silica affected the rubber matrix, resulting in foam rubber with smaller and evenly arranged rubber cells. The hydrophobic fumed silica, Reolosil DM30, with its high specific surface area, influenced the performance of the reinforced rubber foam. A larger BET typically results in a higher contact area between rubber cells and silica, meaning stronger interaction between the silica filler and natural rubber, potentially leading to lower compression set properties. This means that the foam rubber retains its shape better after compression [7,12]. Conversely, a lower specific surface area may reduce the propensity for agglomeration, thus enhancing the compression set properties. At 2 phr, the compression set of Reolosil DM30 was 12.0% ± 1.0%, while Aerosil R972, another hydrophobic fumed silica with a lower BET, had a compression set value of 14.0% ± 0.6% (Figure 9).

Additionally, hydrophilic silica has a higher affinity for water and may retain water molecules within the material. This water absorption can lead to swelling of the rubber matrix, affecting its elastic recovery properties. Consequently, rubber composites containing hydrophilic silica may exhibit higher compression set values due to increased permanent deformation under compression. This is reflected in the results: at 2 phr, Aerosil 200, a fumed silica with hydrophilic nature, had the highest compression set value of 17.9% ± 0.6% because of its poorer and more uneven, porous distribution [48], as shown in Figure 3. Similarly, the compression set of precipitated silica, which is also hydrophilic in nature, had a higher compression set value (14.8% ± 0.8%) than both hydrophobic silicas.

### 3.6. Hardness

**Figure 10:**
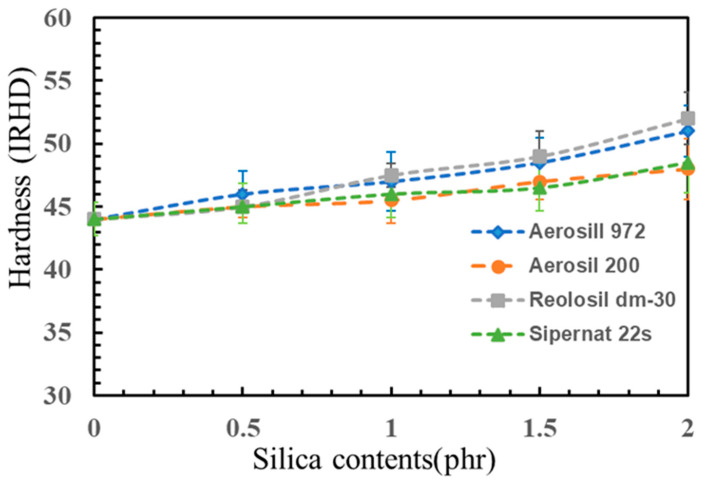
Hardness of different types of silica with different contents.

Increasing the loading of silica in rubber composites typically results in increased hardness, as demonstrated in Figure 10. Non-silica foam exhibited a hardness value of 45.0 ± 1.3 IRHD, and by incorporating silica into the rubber, the hardness of the foams increased accordingly. As silica loading increases, more filler particles disperse throughout the rubber matrix. The addition of silica contributes to reducing the size of bubbles or cells within the foam structure. This reduction in cell size is facilitated by the increased surface area available for interaction between the silica particles and the rubber chains [52]. Consequently, stronger interfacial adhesion forms between the silica particles and the rubber matrix. With higher silica loading, the foam structure allows less space between the rubber cells, leading to a denser foam structure. This densification significantly contributes to increased hardness [50]. By minimizing the space between cells, the foam becomes less compressible and more resistant to deformation.

A comparison of different silica fillers revealed that the hydrophobic silica, particularly Reolosil DM30 and Aerosil R972, exhibited the highest enhancement in rubber properties compared to the hydrophilic silica, Sipernat 22S and Aerosil 200. At a concentration of 2 phr, Aerosil R972 and Reolosil DM30 demonstrated hardness values of 51.0 ± 2.0 IRHD and 52.0 ± 2.1 IRHD, respectively, while Sipernat 22S and Aerosil 200 exhibited hardness values of 48.5 ± 2.4 IRHD and 48.0 ± 2.4 IRHD, respectively. One significant factor contributing to this observation is the BET surface area of the silica fillers. Reolosil DM30, known for its hydrophobic nature, possesses the highest BET surface area among the tested silica fillers. The higher BET surface area allows for a greater number of active sites to be available for interaction with the rubber matrix, promoting stronger filler–matrix adhesion and ultimately resulting in increased hardness [38,53].

### 3.7. Percentage Shrinkage

**Figure 11:**
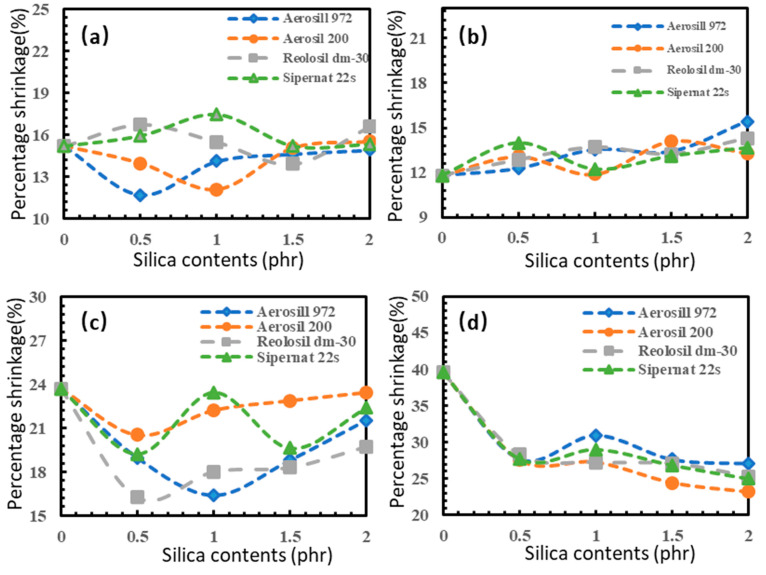
Percentage shrinkage of different types of silica compared with sides of sample: (**a**) width; (**b**) length; (**c**) height; and (**d**) height of center.

Figure 11 depicts the percentage shrinkage of latex foam for four distinct silica concentrations. It is evident that the various silica concentrations resulted in different percentage shrinkage values. The addition of silica led to a notable reduction in the shrinkage of the center height in comparison to non-silica rubber. However, the height and length of the samples exhibited a gradual increase in percentage shrinkage with higher silica loadings. In the width dimension, there was no clear trend, but adding silica loadings at 0.5 and 1 phr could reduce the percentage shrinkage. An intriguing observation was made regarding the height of the center, where the percentage shrinkage exhibited a decreasing trend as the silica loadings increased. The samples’ height and length were smaller and shorter with higher silica loadings, but the center of the samples showed an opposite trend, with the height being greater.

This phenomenon of shrinkage may be attributed to the rubber’s relatively low resistance to swelling in solvents and incomplete foaming rates, resulting in varying densities of the rubber [54]. Furthermore, variations in the quantity of the solids and the types of chemicals employed in the formula, including the foaming agent, can give rise to uncertainties in the mechanical properties of the rubber and affect the percentage of shrinkage [55].

In this experiment, the incorporation of silica as a filler resulted in a reduction in the percentage shrinkage of rubber in the compound. The incorporation of silica as a powder is effectively linked with rubber particles, resulting in a thicker and harder rubber matrix. Consequently, as the silica loadings increased, the shrinkage percentage decreased due to the densification and reinforcement effects of silica on the rubber matrix [56].

## 4. Conclusions

This study investigated the influence of different types and concentrations of silica as fillers, including hydrophilic and hydrophobic fumed silicas, as well as precipitated silica, on the properties of natural rubber foam produced using the Dunlop process. The physical properties, swelling, hardness, and shrinkage behavior of the natural rubber foam were determined. Four different types of silica were added to the rubber formulation with loadings ranging from 0 to 2 phr. The results indicated that the incorporation of silica fillers—both fumed and precipitated—could be evenly distributed in the rubber matrix. Hydrophobic fumed silicas exhibited better dispersion and enhanced the physical and mechanical properties of the natural rubber foam. In the SEM analysis, it was observed that the hydrophobic silicas had a more homogeneous dispersion and integration with the rubber matrix, leading to improved reinforcement and physical/mechanical performance compared to the hydrophilic silica. Even with a lower specific surface area (BET), the hydrophobic fumed silica, Aerosil R972, was still better dispersed compared to hydrophilic silica with a higher BET. Increasing loadings of silica had a noticeable effect on the porosity of the foam rubber; higher silica loadings resulted in smaller foam pores due to an increase in crosslink density as the silica concentration increased, which led to a more consistent pore structure in the foam rubber matrix. By contrast, a lower specific surface area made the average pore diameter smaller, as observed with the hydrophilic fumed silica, Aerosil 200 (29.4 ± 1.7 μm). Higher loadings of silica also increased the foam density and hardness. The compression set decreased as silica loading increased, meaning the foam rubber could recover its original shape better when silica filler was added. Hydrophobic fumed silica with a higher specific surface area, Reolosil DM30, provided the best overall reinforcement and improvement in the properties of natural rubber foam. Even with its lower specific surface area, the hydrophobic fumed silica, Aerosil R972, still resulted in greater hardness and recovery percentages compared to hydrophilic silica. However, excessive silica loading resulted in an increase in the shrinkage of the foam, except for the height of the center foam, which showed a decrease in shrinkage. In this experiment, the Dunlop process was used as the foaming method. Loadings of silica higher than 2 phr were not suitable, as excessive fillers impaired the foaming process and prevented some silica from mixing well with the natural rubber. FTIR spectra analysis was used to try to confirm the chemical interaction between silica and natural rubber. However, the results of the FTIR analysis were not clear enough to identify differences in the chemical bonds between hydrophobic and hydrophilic silica and natural rubber, as the silica loading was too low. It only showed a lower peak of the O-H band in hydrophobic silica compared to hydrophilic silica.

## Figures and Tables

**Table 1:** Formulation of latex foam in parts per hundred of rubber (phr).

Materials	Weight (phr)
High Ammonia concentrated natural latex 60% DRC	100
Potassium-oleate solution 10%	1
Sulphur dispersion 50%	2.5
Zinc-N-diethyldithiocarbamate dispersion 50%	1
Zinc 2-mercaptobenzothiazole dispersion 50%	1
Wing stay L dispersion	1
Zinc Oxide dispersion of 50%	4
Diphenyl guanidine dispersion 50%	1
Sodium silicofluoride dispersion 20%	2.5
Filler dispersion fumed or precipitated silica	0.5, 1, 1.5, 2

**Table 2:** Key characteristics of silica samples used in this study.

Silica Name	Specific Surface Area (m^2^/g)	Carbon Content (%)	Tamped Density (g/cm^3^)	Ph Value (4% Suspension)	SiO_2_ Content (wt.%)	Hydrophilic/Hydrophobic
Aerosil 972	110 ± 20	0.6–1.2	Approx. 50	3.6–5.5	≥99.8	hydrophobic
Reolosil Dm30	235 ± 20	1.7	Approx. 50	4.8	≥99.8	hydrophobic
Aerosil 200	200 ± 25	N/A *	Approx. 50	3.7–4.7	≥99.8	hydrophilic
Sipernat 22s	190	N/A *	Approx. 90	6.5	≥97	hydrophilic

* N/A indicates that the information is not available in the product specifications.

## Data Availability

The original contributions presented in the study are included in the article, further inquiries can be directed to the corresponding authors.
